# Evolution of Northeastern and Midwestern *Borrelia burgdorferi*, United States

**DOI:** 10.3201/eid1606.090329

**Published:** 2010-06

**Authors:** Dustin Brisson, Mary F. Vandermause, Jennifer K. Meece, Kurt D. Reed, Daniel E. Dykhuizen

**Affiliations:** University of Pennsylvania, Philadelphia, Pennsylvania, USA (D. Brisson); Marshfield Clinic Research Foundation, Marshfield, Wisconsin, USA (M.F. Vandermause, J.K. Meece); Northwestern University/Feinberg School of Medicine, Chicago, Illinois, USA (K.D. Reed); Stony Brook University, Stony Brook, New York, USA (D.E. Dykhuizen)

**Keywords:** Bacteria, vector-borne infections, Lyme disease, evolution, incidence, Borrelia burgdorferi, geography, United States, research

## Abstract

The per capita incidence of human Lyme disease in the northeastern United States is more than twice that in the Midwest. However, the prevalence of *Borrelia burgdorferi*, the bacterium that causes Lyme disease, in the tick vector is nearly identical in the 2 regions. The disparity in human Lyme disease incidence may result from a disparity in the human invasiveness of the bacteria in the Northeast and Midwest caused by fundamentally different evolutionary histories. *B. burgdorferi* populations in the Northeast and Midwest are geographically isolated, enabling evolutionary divergence in human invasiveness. However, we found that *B. burgdorferi* populations in the Northeast and Midwest shared a recent common ancestor, which suggests that substantial evolutionary divergence in human invasiveness has not occurred. We propose that differences in either animal ecology or human behavior are the root cause of the differences in human incidence between the 2 regions.

## Abstract 2

Differences in animal ecology or human behavior may account for differences in human incidence in the 2 regions.

Lyme disease, caused by the bacterium *Borrelia burgdorferi*, is the most common vector-borne disease in the United States (*1*). *B. burgdorferi* is transmitted to humans during the blood meal of an infected *Ixodes* tick, predominantly *Ixodes scapularis* in North America (*2*). The prevalence and density of *B. burgdorferi*–infected *I. scapularis* ticks are nearly identical in the northeastern and midwestern United States, the regions with the highest incidence of Lyme disease in humans (*3*–*6*); however, the number of human Lyme disease cases reported in the Northeast and Midwest is not (*1*). The overwhelming majority of Lyme disease cases in the United States are reported from the Northeast (82%), followed distantly by the Midwest (9%) (*1*). Similarly, per capita Lyme disease incidence is >2× greater in the Northeast than the Midwest. We address the hypothesis that *B. burgdorferi* populations in the Northeast and Midwest have fundamentally different evolutionary histories, which may result in differing degrees of human invasiveness.

The evolutionary and demographic histories of *B. burgdorferi* in the Northeast have been intensively studied. The effective population size of northeastern *B. burgdorferi* is small because of its recent colonization of the northern United States and its life-history strategy (*7**,**8*). The strikingly impoverished neutral genetic diversity and high linkage disequilibrium within *B. burgdorferi* populations likely result from small effective population sizes (*9**–**11*). Genetic loci are found in perfect or near-perfect association in *B. burgdorferi* in the Northeast (*9**–**12*). Strong linkage disequilibrium among genetic loci can result from several evolutionary and ecologic forces in addition to small population size (drift), such as lack of recombination machinery or limited opportunity for gene exchange (*13*). Genetically diverse strains of *B. burgdorferi* often are found within the same tick or same vertebrate host, suggesting ample opportunity for genetic exchange (*4**,**14*). Evidence is strong that recombination occurs within a genomic lineage of *B. burgdorferi* (*15**–**17*). Thus, *B. burgdorferi* has the opportunity and the recombination system needed for genetic exchange. A historically small effective population size is a parsimonious explanation for the low neutral genetic diversity and strong linkage disequilibrium.

The evolutionary and demographic histories of *B. burgdorferi* in the Midwest are comparatively understudied. On the coarse scale, the evolutionary history and ecology of *B. burgdorferi* in the Northeast and Midwest appear similar. Both regions have oak-maple–dominated forests ideal for deer and the small mammals that maintain the *I. scapularis* and *B. burgdorferi* populations, were under the Pleistocene ice sheet, and were recently colonized by *B. burgdorferi*. The differences accounting for a lower Lyme disease incidence in the Midwest than the Northeast are not clear but most likely can be found on a finer scale in the evolutionary history of *B. burgdorferi* (this study), the tick vector (*8*), or human exposure.

The differences accounting for a lower Lyme disease incidence in the Midwest than the Northeast are not clear but are likely to be found on a finer evolutionary or ecologic scale. A recent report suggested that a lower proportion of the 16S–23S rRNA intergenic spacer (IGS) type 1 (RST-1) allele in midwestern *B. burgdorferi* populations, an allele associated with human invasiveness in the northeastern United States, may account for the differences in human Lyme disease incidence (*8*). However, the 16S–23S rRNA IGS does not directly influence *B. burgdorferi* invasiveness; it is in linkage disequilibrium with a gene of major effect in the Northeast. The linkage patterns in midwestern *B. burgdorferi* are as yet unstudied. A fundamentally different evolutionary history would result in divergent linkage disequilibrium patterns between the Northeast and the Midwest and potentially result in differing degrees of human invasiveness associated with alleles at the 16S–23S rRNA spacer.

For this study, we used a phylogenetic framework to analyze the evolutionary and demographic histories of *B. burgdorferi* in the midwestern and northeastern United States (*18*). A previous multilocus sequencing typing study of 4 *B. burgdorferi* loci (outer surface protein C [*ospC*], 16S–23S rRNA (*rrs-rrl-A*), *ospA*, and outer membrane protein [*p66*]) from strains isolated in the Northeast identified 9 distinct lineages with complete linkage among alleles at the 4 loci (*10*). That study analyzed statistical associations of haplotypes at each locus without regard to the underlying evolutionary relationships of sequences. In this study, we analyzed *ospC*, *rrs-rrlA*, *ospB*, and *ospA* (p66 contains little evolutionary information [*10*]) from midwestern and northeastern populations in a phylogenetic framework to investigate the shared and vicariant evolutionary and demographic histories of *B. burgdorferi* from geographically isolated regions.

## Methods

### *B. burgdorferi* Isolates

All isolates were derived from skin biopsy specimens of 47 adult patients who had erythema migrans, at the Marshfield Clinic in central Wisconsin during 1995–2001. Specimens were collected and cultured as described elsewhere (*19*).

### DNA Extraction and Amplification

The DNA sequences of *ospA*, *ospB*, *rrs-rrlA*, and *ospC* were determined for use in our phylogenetic analysis. Cultivated isolates were harvested by centrifugation at 5,000 × *g* for 15 min, resuspended in sterile water, and lysed by boiling for 5 min. PCR conditions are described by Bunikis et al. (*10*) for *ospA* and *rrs-rrlA*, by Caporale and Kocher (*20*) for *ospB*, and by Brisson and Dykhuizen (*4*) for *ospC*. Negative controls were included for DNA extraction and PCR procedures to monitor for contamination. Amplified PCR products were sequenced in both directions. *ospC* products were subject to the reverse-line blot procedure as described previously (*4**,**7*). *ospC* amplicons that could not be classified to a major allelic group were sequenced.

### Analyses

All analyses included sequence data collected in this study and reported in Bunikis et al. (*10*)*.* DNA sequences were aligned using the Clustal X algorithm (*21*) with default settings, and alignments were refined manually where necessary. Descriptive statistics (π, synonymous and nonsynonymous polymorphisms) were determined using DNAsp (ver. 4.50.3) (*22*). Two tests for recombination within genes (Sawyer test and maximum χ^2^ test) and a test for allelic association (index of association [I_A_]) were performed in START2 (*23*). Sawyer runs test compares pairs of alleles to determine whether regions of the sequence space have more consecutive identical polymorphisms (runs) than expected by chance (*24*). The maximum χ^2^ test uses the distribution of polymorphic sites to identify potential recombination events between pairs of alleles (*25*). The I_A_ assesses the extent of association between loci using allele frequencies without regard to the underlying sequences (*26*).

Phylogenetic reconstruction was performed using Bayesian (MrBayes; http://mrbayes.csit.fsu.edu) and maximum likelihood (PAUP*, version 4.0; http://paup.csit.fsu.edu) approaches. Trees were constructed from the sequence data from each gene individually and from combinations of genes. IGS, *ospA*, and *ospB* provide reliable data for phylogenetic inference and can be easily compared with previous analyses (*10*). *ospC* DNA sequences provide information about disease invasiveness (*27**,**28*) but were not used in phylogenetic reconstruction because the gene tree topology is star-shaped with short and unsupported internal branches, long terminal branches, and many polytomies. This topology provides no information about the evolutionary history of this gene or the relationships among alleles. When information about *ospC* was included in the data set, it was included as a heavily weighted morphologic character in MrBayes, not as DNA sequence data. The morphologic data constrain the isolates with the same *ospC* allele cluster but do not affect the topology of the internal branches of the tree, which depend only on the DNA sequences of the other genes in the data set. For each data set, we used Modeltest 3.4 (*29*) to select the appropriate model of molecular evolution. We used this model to find the Bayesian tree and the maximum likelihood tree.

We used the Shimodaira-Hasegawa (SH) test as implemented in PAUP to test for differences in evolutionary histories among genes using the resampling of estimated log-likelihoods approximation with 1,000 bootstrap replicates (*18*). This method tests for significant differences in the likelihood of several given tree topologies given a data set. The likely tree topologies produced by each locus analyzed in this study were used as the set of topologies. If no true difference exists, each data set is equally likely to produce any of the given tree topologies, and all genes in this study share similar evolutionary histories. Alternatively, a significantly lower likelihood suggests that recombination has occurred between the genes in the data set and the gene used to create the lower-likelihood tree topology.

## Results

### Sequence Diversity

*B. burgdorferi* isolates were cultured from primary or secondary skin legions of 47 adults visiting the Marshfield Clinic during 1995–2001. The sequences at the IGS, *ospA*, *ospB*, and *ospC* loci were determined from 39, 31, 44, and 44 strains of these *B. burgdorferi* isolates, respectively. Erythema migrans represented the bulk of the diversity found in tick samples (*27*) and provided a representative sample of *B. burgdorferi* bacteria in the Midwest. Furthermore, we found all of the IGS types found by Bunikis et al. (*10*), suggesting the sample from the Midwest was not biased in terms of the types found. *ospA* showed little evolutionarily information and was not determined from 14 of the isolates. One sequence was found at each locus in each culture.

Nucleotide diversity varied considerably among loci (Table 1). We found 47 polymorphic sites in the IGS sequence sample, resulting in 16 unique haplotypes (Technical Appendix). Six haplotypes were identical to haplotypes previously found in the Northeast, and 38 of the 47 polymorphic sites found in the Northeast were present in this sample from the Midwest, supporting a recent shared ancestry of *B. burgdorferi* from the 2 populations (Technical Appendix). The 10 unique haplotypes in the Midwest and 9 unique polymorphic sites suggest some isolation by distance and an emerging evolutionary divergence.

**Table 1:** Genetic markers and population diversity of *Borrelia burgdorferi*, midwestern and northeastern United States, 1999–2001*

Marker	No. samples	π	dN/dS	Maximum χ^2^, p value	Sawyer test, significant fragments
IGS	39	0.014	NA	107, p<0.01	0
*ospA*	31	0.0033	0.334	0	0
*ospB*	44	0.0042	0.303	739, p<0.05	0
*ospC*	44	0.207	0.591	74, 83, 90, 137, 338; p<0.001	4

*dN/dS, directional selection; IGS, intergenic spacer; NA, not applicable; osp, outer surface protein.

*ospA* and *ospB* are considerably less diverse than IGS (Table 1). Nevertheless, there are 14 unique *ospA* haplotypes and 14 unique *ospB* haplotypes in this sample (Technical Appendix). Nine of the *ospA* haplotypes in the samples from the Midwest also are found in *B. burgdorferi* in the Northeast. The diversity of *ospB* has not been examined in the Northeast. Amino acid sequence evolution in *ospA* and *ospB* appears to be constrained because synonymous changes greatly exceed nonsynonymous changes (dN*_ospA_*/dS*_ospA_* = 0.334; dN*_ospB_*/dS*_ospB_* = 0.303).

The mean pairwise diversity at *ospC* was much greater than at the other loci analyzed (π = 0.207). Sixty-three changes resulted in amino acid substitutions. However, we found no evidence of directional selection (dN/dS = 0.591). Eighteen major groups of alleles, which differ by >8% in sequence, are represented in the isolates from the Midwest (Appendix Table). Fourteen of the 18 major group alleles are identical to those found in the Northeast, although types L and O are rare in the Northeast. We found 4 novel *ospC* major group alleles (W, V, Y, Z) in these samples (Appendix Table). *ospC* major groups W, V, and X also were detected in tick populations in the Midwest (D. Brisson, unpub. data), although we did not see type X in this set of human isolates. Strains with *ospC* major groups I, J, and T, which are present in tick and human samples in the Northeast, were absent in this sample from the Midwest.

### Phylogenies

We used Bayesian and likelihood algorithms to reconstruct phylogenies for each gene. The 2 approaches resulted in identical topologies for each gene except *ospC.* The IGS gene tree forms a strongly supported phylogeny with most nodes bifurcating (Figure). Polychotomies occur only at sequences that are identical or differed by 1 nucleotide. The phylogeny generally supports the previously described divisions of IGS sequences into the 3 restriction fragment length subgroups, arbitrarily called RST 1, RST2, and RST3 (*31*). However, the diverse RST3 group is not monophyletic. The IGS sequences from strains in the Northeast and Midwest are interdigitated, suggesting a recent shared history.

**Figure:**
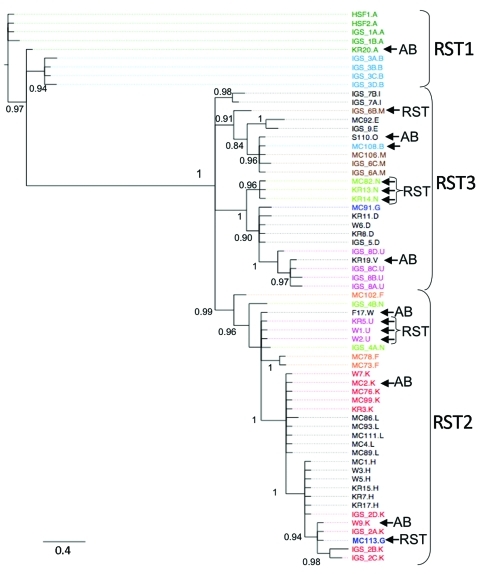
Phylogeny of *Borrelia burgdorferi* isolates in the northeastern and midwestern United States based on intergenic spacer (IGS) sequence. operational taxanomic unit names beginning with IGS were isolated in the northeastern United States (*10*); all other isolates are from patients in the Midwest. The letter after period designates the outer surface protein C (*ospC*) major allele of the isolate. Colored isolate names highlight isolates with the same *ospC* major group that cluster in different clades, which suggests horizontal gene transfer. The *ospC* of several strains is not linked to the IGS ribosomal spacer type (RST) to which it is commonly linked in the Northeast (*10*,*34*). AB indicates differences between the *ospAB* tree and the IGS tree. This tree is midpoint rooted. Scale bar indicates number of substitutions per site.

The gene trees for *ospA* and *ospB* individually have many weakly supported nodes and polychotomies because of the limited number of phylogenetically informative sites. The phylogeny reconstructed from the *ospA/ospB* operon (concatenated data set) result in much stronger support for internal nodes. Nevertheless, the *ospAB* phylogeny contains 1 polychotomy. Concatenating the data sets for *ospA* and *ospB* sequences is appropriate because these loci lie contiguously in a single operon, and little evidence exists for recombination either within or between these genes (see below). The northeastern and midwestern strains were interdigitated on the *ospA*, *ospB*, and *ospAB* trees, which suggests a recent, shared population history.

Analysis of the *ospC* data set yielded a phylogeny with short internal branches, long terminal branches, and no supported internal nodes, commonly referred to as a star phylogeny (*10**,**13*). *ospC* thus appeared to have evolved by recombination (*9**,**32*). Phylogenies of highly recombining loci are poorly supported because the evolutionary history differed in different segments of the allele (*33*).

### Intragenic Recombination

We found little evidence of recombination within IGS, *ospA*, or *ospB* in the Midwest data set or the combined northeastern/midwestern data set (Table 1). Sawyer runs test found no runs of polymorphisms that are significantly incongruous within the sequence. Maynard Smith’s maximum χ^2^ test found 1 recombination event within the IGS sequence at nt 107 (position compared with B31 type strain) in both data sets. We found no evidence for recombination within *ospA* and only 1 potential site of recombination at the extreme 3′ end of *ospB*. Neither maximum χ^2^ nor Sawyer test found evidence for recombination between *ospA* and *ospB* in the *ospAB* operonic data set. However, *ospA* haplotypes are often associated with multiple *ospB* haplotypes suggesting recombination or independent evolution (Technical Appendix). Intragenic recombination was evident within *ospC* sequences. Sawyer test identified 4 runs of polymorphisms that appear to be transferred from another *ospC* major allelic group. Maynard Smith maximum χ^2^ test found evidence of recombination at 5 locations along the sequence (Table 1).

### Intergenic Recombination

We assessed intergenic recombination using the I_A_ and the SH test. The index of association detects nonrandom associations of haplotypes among genes but does not account for sequence variation or relatedness. The I_A_ indicated that all loci examined in this study were significantly nonrandomly associated, i.e., in linkage disequilibrium (p<0.0001) (Table 1).

The SH test uses phylogenetic information to identify horizontal transfer events by comparing the evolutionary histories of genes as represented by the gene-tree topologies. The likelihood of the IGS sequence data resulting in the *ospAB* tree topology is significantly less likely than these data resulting in the IGS topology (Table 2). That is, the evolutionary history of the IGS locus is not congruent with the *ospAB* evolutionary history, providing evidence of lateral gene transfers. Strains found in different strongly supported clades on the *ospAB* and the IGS trees are identified in the Figure (p<0.0001).

**Table 2:** Summary of Shimodaira-Hasegawa test results of potential horizontal gene transfer events in *Borrelia burgdorferi*, midwestern and northeastern United States*

Test comparison	Data set	Δ lnL	p value
*ospAB* vs.			
IGS	*ospAB*	8.50709	0.390
*ospAB/ospC*	*ospAB*	21.26004	0.313
IGS vs.			
*ospAB*	IGS	642.826545	<0.0001
IGS/*ospC*	IGS	67.53033	0.006
IGS/*ospC*†	IGS	388.21228	<0.0001
*ospB* vs. *ospB/ospC*	*ospB*	18.91326	0.338
*ospA* vs. *ospA/ospC*	*ospA*	45.30135	0.146
*ospA* vs. *ospA/ospC*†	*ospA*	79.62589	0.080

*Δ InL, difference in log-likelihood; *osp*, outer surface protein; IGS, intergenic spacer. †Included sequence data from Bunikis et al. (*10*).

The phylogenies reconstructed from midwestern *ospAB* sequences were compared with *ospAB* phylogenies that constrain strains with the same *ospC* allele to cluster (*ospABC* tree). We found no statistical support for recombination between *ospC* and *ospAB* (p = 0.313). However, 4 *ospC* type K strains (W9, W7, MC2, KR3) are separated from the other K strains by supported nodes in the *ospAB* tree, and 1 *ospC* type F strain (MC102) is separated from the other *ospC* type F strains. We also found no evidence of recombination between *ospA* and *ospC* or *ospB* and *ospC* (p>0.1). However, the *ospA* tree is nearly significantly more likely than the *ospAC* tree when strains from the Northeast are included in the analysis (p = 0.08).

Several instances of recombination are evident between IGS and *ospC*. The differences in evolutionary history were observable by using the midwestern IGS data set alone (p = 0.006) and the combined midwestern/northeastern IGS data set (p<0.001). These analyses provide evidence that linkage patterns in *B. burgdorferi* from the Northeast differ from that of the linkage patterns in *B. burgdorferi* from the Midwest.

## Discussion

The divergence in human Lyme disease incidence between the Northeast and Midwest does not result from independent evolution of human invasiveness because of geographic isolation. Although pathogen populations in the Midwest appear geographically isolated from those in the Northeast (*6*), evolutionary and demographic analyses indicate that they share a recent common ancestor. Both populations have little standing genetic variation, as indicated by the limited number of polymorphic sites, suggesting small effective population sizes and similar life-history strategies. The combination of linked alleles also is similar in both regions, supporting the recent shared ancestor hypothesis. *B. burgdorferi* strains isolated in the Midwest are interleaved with northeastern strains on phylogenetic trees evincing their close evolutionary relationship. However, there is some genetic divergence and differing linkage groups between the regions, intimating that gene flow is limited between these populations, allowing them to differentiate. The recent common ancestor in the northeastern and midwestern *B. burgdorferi* and limited genetic divergence suggests that human Lyme disease incidence cannot be explained by fundamentally different evolutionary histories resulting in differing degrees of human infectiousness.

None of the loci investigated show substantial genetic divergence between regions, suggesting a recent common ancestor and similar phenotypes. Northeastern haplotypes are interleaved with midwestern haplotypes such that the time to coalescence of alleles within a region is equivalent to the time to coalescence for alleles from both regions (Figure). These data suggest that northeastern and midwestern strains have a recent common ancestor. The limited genetic diversity in *B. burgdorferi* in the Midwest and Northeast (Technical Appendix) suggests that the populations have retained the life-history strategy of their common ancestor (*10**,**12*). However, isolation by distance and subsequent divergence resulted in unique alleles in each region.

The IGS gene tree reconstructed from midwestern and northeastern data broadly supports the RST system described using northeastern populations (*31*). RST types 1 and 2 form strongly supported monophyletic groups. RST3 is polyphyletic and should be split into 3 groups as defined by the strongly supported clades (Figure). Supporting this suggestion, RST3 is diverse genetically and phenotypically (*34*). Interestingly, this division would separate *ospC* major group I bearing strains, a particularly invasive group in humans, from the other RST3 strains that rarely cause disseminated infections in humans (*27**,**28**,**30*).

The *ospC* data support the hypothesis that the strains from the Northeast and Midwest have a common ancestor but are currently isolated and have begun to diverge. Most *ospC* major groups are found in both regions. Given the genetic distance between major group alleles, the exact set of alleles is unlikely to have occurred twice independently. Additionally, the linkage relationships between *ospC* alleles and IGS alleles are similar in both regions. Both lines of evidence suggest that most of the diversity at *ospC* originated before the northeastern and midwestern populations diverged. Differences in invasiveness between *B. burgdorferi* in the Northeast and Midwest do not result from fundamentally different evolutionary histories.

Four novel *ospC* major group alleles appear to be unique to the Midwest (Table 2). In addition to these novel *ospC* major groups, a type C–like allele appears to have been generated independently in the Midwest and the Northeast. The group C allele in the Midwest shares 96.8% similarity with the group C allele in the Northeast. Whether the unique *ospC* major group alleles were generated recently in only 1 region or whether they were shared in the ancestral population and subsequently lost in only 1 region is not clear.

*B. burgdorferi* lineages exchange DNA, contrary to previous reports (*10**,**11**,**13*). We found at least some evidence of recombination between all genetic loci examined; even the *ospAB* operon has several homoplasious mutations, suggesting potential recombination (Technical Appendix). However, recombination between *ospA* and *ospB* is not statistically supported and may have arisen from recurrent mutation (Appendix Table). Despite evidence for recombination, the linkage relationships are similar in the Northeast and the Midwest, supporting the recent common ancestry of these populations.

Recombination is more apparent in Lyme disease foci in the Midwest than in the Northeast (Figure; Appendix Table). This is likely to be caused by neutral divergence of linkage patterns resulting from small effective population sizes in both regions coupled with gene flow from the Northeast to the Midwest but not in the other direction. Small effective population sizes eliminate most of the linkage combinations in each region such that they are in perfect linkage disequilibrium. Gene flow from the northeastern population then introduces linkage pattern variation into the midwestern population. Linkage patterns unique to the Midwest are absent from the Northeast, suggesting that gene flow from the Midwest to the Northeast is rare.

*B. burgdorferi* in the Northeast and Midwest share a remarkably similar evolutionary history. Independent evolution of human invasiveness in the 2 regions does not explain the lower human Lyme disease incidence in the Midwest. Other potential causes for the differences in human Lyme disease incidence include differences in human exposure to *B. burgdorferi*–infected ticks and ecologic differences in the reservoir host community. Lyme disease typically is contracted peridomestically in the Northeast (*35*), but similar studies reporting the peridomestic acquisition of *B. burgdorferi* have not been reported from the Midwest. Human risk for exposure to Lyme disease also may be exaggerated in the Northeast because of the immense suburban populations around the major metropolitan areas. Current ecologic conditions yielding differences in the composition of reservoir host species could alter the prevalence of *B. burgdorferi* lineages that are particularly invasive in humans (*27**,**28**,**30**,**36**,**37*). For example, midwestern ticks are rarely infected with *ospC* genotypes A or B (RST I) (*3*), 2 of the 4 genotypes that are common in the Northeast and regularly cause human Lyme disease (*27**,**28**,**30*).

## Supplementary Material

Appendix Table Distance matrix of DNA sequences from the outer surface protein Cgene of Borrelia burgdorferi*

Technical Appendix Statistical Support for Horizontal Gene Transfer
