# Supplementary material for: Evolution of Northeastern and Midwestern Borrelia burgdorferi, United States
Source: Emerg Infect Dis. 2010 Jun;16(6):911–7. doi: 10.3201/eid1606.090329 (PMC3086229; doi:10.3201/eid1606.090329)
Supplement: Appendix Table — Distance matrix of DNA sequences from the outer surface protein Cgene of Borrelia burgdorferi* [file 09-0329-appT-s1.pdf]

Appendix Table. Distance matrix of DNA sequences from the outer surface protein Cgene of Borrelia burgdorferi*

Sequence	A	B	C	C_{KR10}	D	E	F	G	H	I	J	K	L	M	N	O	P	Q	R	S	T	U	V_{MM25}†	W_{F17}	X_{WI30}	Y_{BC2}	Z_{KR18}
A																											
B	79.8																										
C	83	84.3																									
C_{KR10}	81.9	84.7	96.8‡																								
D	84.5	79.9	79.1	78.6																							
E	78.5	77.8	79.8	78.5	76.3																						
F	78.3	76.1	80.4	78.7	79.8	78.1																					
G	79.8	76.6	76.8	75.7	78.5	80.1	78.5																				
H	78.5	74.4	81.1	80.2	77.6	77	82.8	75.9																			
I	81.7	80.2	87.7	86.5	81.6	79	79.6	74.8	80.2																		
J	81.3	76.8	83.2	82.2	81.9	78.5	84.5	78.5	89.7	82.4																	
K	82.4	78.7	83	81.9	81.5	81.5	83	78.3	81.5	82.6	83.7																
L	74.2	78.5	77.2	77.4	76.1	75.9	76.6	73.8	80.9	79.8	80.6	76.3															
M	80.4	74.6	79.1	77.6	78.1	81.3	81.1	77.4	76.3	78.7	78.7	81.5	75.1														
N	76.1	79.8	79.1	78.7	77	76.8	76.6	82	75.3	77	77.2	79.6	74.6	76.4													
O	75.9	83.3	80.4	80	78.7	81.3	80.9	78.1	75.3	77.4	77	77.2	78.5	80.4	78.5												
P	77.8	76.3	76.3	75.9	75.7	79.4	79.6	83.7	75.7	75.5	79.1	76.6	74.2	78.5	78.5	76.1											
Q	81.1	76.6	84.3	83.7	77.8	77.4	80.2	76.8	83	84.5	82.2	80.2	77.2	78.1	77.6	77	76.6										
R	77.4	74.2	77.6	75.9	76.1	78	78.3	82.4	75.1	77.4	77.6	78.5	73.5	79.8	77.6	77.4	83.7	75.3									
S	78.7	82.6	80.4	79.4	79.4	79.1	77.6	81.3	75.6	78.1	79.2	78.3	77	76.6	79.4	81.8	79.8	75.9	82								
T	78.9	78.9	77.6	76.8	78.3	76.4	76.1	80.4	72.7	77.2	75.1	78.5	74.9	77	78.3	77.5	80.2	74.8	76.1	80							
U	79.8	76.8	78.3	77	74.6	74.2	77.1	81.7	74.8	75.3	76.8	74.8	76.3	75.9	76.7	78.6	81.1	75.2	80.9	84.1	83.2						
V_{MM25}‡	83.1	78.3	85.5	84.1	83.2	85.1	86.5	82.1	87.9	86	89.4	90.8	81.6	87	82.2	82.1	80.7	85.5	83.6	80.2	78.8	78.7					
W_{F17}	79.4	79.4	82.4	82.2	78.2	79.6	78.9	77.6	75.5	81.7	79.6	79.6	80	84.1	77	81.1	77.2	78.3	77.6	79.8	76.6	78.3	84.1				
X_{WI30}	82.6	76.8	82.6	80.9	80.9	76.8	79.6	79.1	78.9	80.2	82.2	81.3	75.7	78.5	77.8	79.4	77.2	79.1	80.4	79.8	76.6	78.7	86.5	78.1			
Y_{BC2}	75.9	77.4	78.3	78.5	75.7	79.1	78.9	75.9	79.4	76.1	79.6	78.1	81.9	78.7	75.5	80	77.8	79.1	75.7	79.3	76.8	78.7	86	78.9	75.5		
Z_{KR18}	77.4	77.4	80	79.4	78.9	77.6	79.3	77.6	76.5	77	78.9	82.6	74.4	78.7	78.8	77	77.2	75.9	78	77.6	75.7	75.3	86	76.6	76.3	75.5	

*Used only first 300 bp, compared with NC_001903. GenBank accession nos HM047873, HM047874, HM047875, HM047876, HM047877, and HM047878.
†Strain designations..

‡KR10 is >2% and <8% different from C; <2% means the same allelic group, >8% means a different group.
