# Supplementary material for: Evolution of Northeastern and Midwestern Borrelia burgdorferi, United States
Source: Emerg Infect Dis. 2010 Jun;16(6):911–7. doi: 10.3201/eid1606.090329 (PMC3086229; doi:10.3201/eid1606.090329)
Supplement: Technical Appendix — Statistical Support for Horizontal Gene Transfer [file 09-0329-Techapp-s2.pdf]

Evolution of Northeastern and Midwestern *Borrelia burgdorferi*, United States

Technical Appendix

Statistical Support for Horizontal Gene Transfer

Several instances of recombination are evident between intergenic spacer (IGS) and the outer surface protein C (*ospC*) gene. Differences in evolutionary history are observable by using the midwestern IGS data set alone ($p = 0.006$) and the combined midwestern/northeastern IGS data set ($p<0.001$). The different p values in the 2 analyses could result simply from different sample sizes used in the Shimodaira-Hasegawa test (63 strains in the midwestern/northeastern data set; 39 strains in the midwestern data set). We constructed 10 data sets by randomly sampling 39 strains from the midwestern/northeastern data set. The constrained and unconstrained phylogenies were reconstructed and compared for each randomly sampled data set. The topologies of the unconstrained trees from all the random data sets were significantly more likely than constrained trees, supporting evidence of lateral gene transfer. Moreover, the p values were <0.006 in 9 of the 10 cases, suggesting that including northeastern linkage relationships increases differences between constrained and unconstrained trees ($p \leq 0.001$, <0.001, <0.001, 0.001, 0.002, 0.002, 0.004, 0.004, 0.006, 0.008), despite the perfect linkage disequilibrium between IGS and *ospC* in the northeastern United States.

Technical Appendix Table 1. Midwestern and northeastern IGS haplotypes

Strain	Alignment<br>11111222222222223333344444444445555555555666666666677 7<br>1346777777778899000011224557888833346113368889012347788801225567889111<br>6134134567893658267998698050235626959594568235065551602903463622245136	Midwest frequency	Bunikis type*
Hap_1	ACAAA-------AGTTAGGTTCTATAGGACAGAGTCCTCACGGACTGGTACTTCGGGTCCAATCACTCCC	2	1A
Hap_2	.....-------...T.T....	1	
Hap_3	.....-------................G...	0	1B
Hap_4	.....-------...G............G..................................T.TC...	0	3C
Hap_5	.....-------.-.G............G..................................T.T....	0	3A
Hap_6	.....-------...G............G..................................T.T....	0	3B
Hap_7	.....-------...G............G........C.........................T.T....	0	3D
Hap_8	..TGGATTTTTTTAGCGAA.GT..C..A.GGAGC...C...A.G..ATCG..C.T.....C.CTGT....	3	6A
Hap_9	..TGGATTTTTTTAGCGAACGT..CG.A.GG.GC...C...A.GA.ATCG.CC.T.....C.CTGT....	1	
Hap_10	G.TGGATTTTTTTAGCGAA.GTCG.....GG.GC...CT..A.G.CA.CG..C.T.....C.CTGT....	1	
Hap_11	G.TGGATTTTTTTAGCGAA.GTC......GG.GC...CT..A.G.CA.CGT.C.T.....C.CTGT....	1	
Hap_12	GTTGGATTTTTTTAGCGAA.GTC......GG.GC..TCT..A.G.CA.CG..C.T...T.C.CTGT....	1	8C
Hap_13	G.TGGATTTTTTTAGCGAA.GTC......GG.GC...CT..A.G.CA.CG..C.T.....C.CTGT....	2	5
Hap_14	G.TGGATTTTTTTAGCGAA.GTC.CG...GG.GC...CT..A.G.CA.CG..C.T.....C.CTGT....	3	
Hap_15	..TGGATTTTTTTAGCGAA.GT..C.A..GGAGC...C...A.G..A.CG..CTT.....C.CTGT....	1	
Hap_16	..TGGATTTTTTTAGCGAA.GT..C.A..GG.GCC..C..T..G..A.CG..CTT..C..C.CTGT....	2	
Hap_17	..TGGATTTTTTTAGCGAA.GT..C.A..GG.GC...C.....G..A.CG..CTT..C..C.CTGT....	3	
Hap_18	G.TGG-------TAGCGAA.GT..C.A..GG.GC...C....TG..A.CG..CTT..C..CCCTGT.T..	1	
Hap_19	G.TGG-------TAGCGAA.GT..C.A..GG.GC...C....TG..A.CG..CTT..C..C.CTGT.T..	9	
Hap_20	G.TGG-------TAGCGAA.GT..C.A..GG.GC.T.CT...TG..A.CG..CTT..C..C.CTGT.TT.	2	2A
Hap_21	G.TGG-------TAGCGAA.GT..C.A..GG.GC...CT...TG..A.CG..CTT..C..C.CTGT.TT.	6	2D
Hap_22	G.TGG-------TAGCGAA.GT..C.A..GG.GC.T.CTC..TG..A.CG..C.T..C....CTGT.TT.	0	2B
Hap_23	G.TGG-------TAGCGAA.GT..C.A..GG.GC.T.CT...TG..A.CG..CTT..C.....TGT.TT.	0	2C
Hap_24	..TGGATTTTTTTAGCGAA.GT..C.A..GG.GC...C.....G..A.CG..CTT..C..C.CTGT...T	0	4A
Hap_25	..TGGATTTTTTTAGCGAA.GT..C.A..GG.GC...C.C.A.G..A.CG..CTT..C..C.CTGT...T	0	4B
Hap_26	GTTGGATTTTTTTAGCGAA.GTC......GG.GC..TC...A.G.CA.CG..C.T...T.C.CTGT....	0	8B
Hap_27	GTTGGATTTTTTTAGCGAA.GTC......GG.GC..TCT..A.G.CA.CG..C.T.T.T.C.CTGT....	0	8A
Hap_28	GTTGGATTTTTTTAGCGAA.GTC......GG.GC...CT..A.G.CA.CG..C.T...T.C.CTGT....	0	8D
Hap_30	..TGGATTTTTTTAGCGAA.GT..C....GG.GC...C...A.G..A.CG..C.TT...TC.CTGTC...	0	7B
Hap_31	..TGGATTTTTTTAGCGAA.GT..C....GG.GC...C...A.G..A.CG..C.TT...TC.CTGT....	0	7A
Hap_32	..TGGATTTTTTTAGCGAA.GT..C..A.GG.GC...C...A.G..ATCG..C.TT...TC.CTGT....	0	6B
Hap_33	..TGGATTTTTTTAGCGAA.GT..C..A.GGAGC...CT..A.G..ATCG..C.T.....C.CTGT....	0	6C
Hap_34	..TGGATTTTTTTAGCGAA.GT..CG.A.GG.GC...C...A.G.CATCG.CC.T.....C.CTGT....	0	9

*As defined in Bunikis et al. (*1*).

Technical Appendix Table 2. *ospAB* haplotypes*

Strain	*ospA* ‖ *ospB*<br>2344771222333455777<br>258805551467666917134<br>989762891232069407392	A	B	C	D
Hap_1	GACGTCAACTTTGGGCGTTGA	2	1	1	1†
Hap_2	...A.............G.TC	1	2	2	2
Hap_3	...A.....C.CAA...G..C	1	2	2	3
Hap_4	...AC....C.CAA...G..C	1	3	3	3
Hap_5	...AC....C..A....G..C	1	3	3	7
Hap_6	...ACTC...C.AA...GC.C	1	4		5
Hap_7	.C.AC....C.CAA...G..C	7	5		3
Hap_8	.C.AC....C..A....G..C	1	5		7
Hap_9	...A...G..C.AA...GC.C	1	6		5†
Hap_10	...A...G.C..A....G..C	1	6		7
Hap_11	...A...G.CC.AA.T.G...	1	6		11
Hap_12	.C.ACTC..C..A....G..C	2	7		7
Hap_13	.C.A.TC..C.CAA...G..C	1	8	8	3†
Hap_14	.C.A.TC..C..A....GC.C	3	8	8	8†
Hap_15	.C.A.T...C..AAA.AG..C	2	9	9	6†
Hap_16	.CTA...G....A....G..C	1	10		4
Hap_17	.C.AC.C.AC..AA...G..C	1	11		10
Hap_18	...A.TC..C..A....G...	2	12		9
Hap_19	CC.A.TC..C..A....G...	1	13		9†

*A, frequency in midwestern patients; B, *ospA* haplotypes; C, *ospA* haplotypes as defined in Bunikis et al. (*1*); D, *ospB* haplotypes.
†In the 13 isolates from which *ospA* was not sequenced, 1 *ospB* haplotype 1 and 3 were found, 2 *ospB* haplotype 6 were found, and 3 *ospB* haplotypes 5,8, and 9 were found.

References

1. Bunikis J, Tsao J, Berglund J, Fish D, Barbour AG. Sequence typing reveals extensive strain diversity of the Lyme borreliosis agents *Borrelia burgdorferi* in North America and *Borrelia afzelii* in Europe. Microbiology. 2004;150:1741–55. Medline DOI: 10.1099/mic.0.26944-0
